# Validation of the Withings ScanWatch as a Wrist-Worn Reflective Pulse Oximeter: Prospective Interventional Clinical Study

**DOI:** 10.2196/27503

**Published:** 2021-04-26

**Authors:** Romain Kirszenblat, Paul Edouard

**Affiliations:** 1 Withings Issy-Les-Moulineaux France

**Keywords:** connected watch, COPD, COVID-19, neural network, pulse oxygen saturation, reflective pulse oximeter, sleep apnea syndrome, SpO_2_, Withings ScanWatch, wearable, respiratory, oxygen, respiratory disease, oximeter, validation, accuracy, safety

## Abstract

**Background:**

A decrease in the level of pulse oxygen saturation as measured by pulse oximetry (SpO_2_) is an indicator of hypoxemia that may occur in various respiratory diseases, such as chronic obstructive pulmonary disease (COPD), sleep apnea syndrome, and COVID-19. Currently, no mass-market wrist-worn SpO_2_ monitor meets the medical standards for pulse oximeters.

**Objective:**

The main objective of this monocentric and prospective clinical study with single-blind analysis was to test and validate the accuracy of the reflective pulse oximeter function of the Withings ScanWatch to measure SpO_2_ levels at different stages of hypoxia. The secondary objective was to confirm the safety of this device when used as intended.

**Methods:**

To achieve these objectives, we included 14 healthy participants aged 23-39 years in the study, and we induced several stable plateaus of arterial oxygen saturation (SaO_2_) ranging from 100%-70% to mimic nonhypoxic conditions and then mild, moderate, and severe hypoxic conditions. We measured the SpO_2_ level with a Withings ScanWatch on each participant’s wrist and the SaO_2_ from blood samples with a co-oximeter, the ABL90 hemoximeter (Radiometer Medical ApS).

**Results:**

After removal of the inconclusive measurements, we obtained 275 and 244 conclusive measurements with the two ScanWatches on the participants’ right and left wrists, respectively, evenly distributed among the 3 predetermined SpO_2_ groups: SpO_2_≤80%, 80%<SpO_2_≤90%, and 90%<SpO_2_. We found a strong association and a high level of agreement between the measurements collected from the devices, with high Pearson correlation coefficients of *r*=0.944 and *r*=0.954 on the correlation plots, low Pearson correlation coefficients of *r*=0.083 (*P*=.17) and *r*=0.23 (*P*=.001) on Bland-Altman plots, biases of 0.98% (95% CI 0.65-1.32) and 1.56% (95% CI 1.24-1.87), and root mean square errors of 2.97% and 3.00% from the participants’ right and left hands, respectively.

**Conclusions:**

In conclusion, the Withings ScanWatch is able to measure SpO_2_ levels with adequate accuracy at a clinical grade. No undesirable effects or adverse events were reported during the study.

**Trial Registration:**

ClinicalTrials.gov NCT04380389; http://clinicaltrials.gov/ct2/show/NCT04380389

## Introduction

### Prevalence of Respiratory Diseases

According to the World Health Organization, respiratory diseases are medical conditions affecting the airways and other structures of the lungs. Three of these include chronic obstructive pulmonary disease (COPD), sleep apnea syndrome (SAS), [1] and COVID-19 [2]. COPD is a chronic inflammatory lung disease that causes persistent and progressive airflow limitation [3,4]. Patients with COPD often experience dyspnea, cough, sputum production, and exacerbation, defined as a worsening of the previously cited symptoms. The global prevalence of COPD in 1990 was 10.7% of persons aged at least 30 years, with 227.3 million cases, and it rose to 11.7% in 2010, with 384 million reported cases. By 2030, COPD is predicted to be one of the most common specific causes of death worldwide [5]. COPD is generally caused by tobacco smoke and exposure to outdoor and indoor pollution [3]. COPD is not a life-threatening condition but is associated with multiple comorbidities, such as cardiovascular diseases, hypertension, diabetes mellitus, and osteoporosis, all of which increase the risk of mortality [4,6]. SAS is a sleep disorder in which pauses in breathing or periods of shallow breathing during sleep occur more often than normal [1]. Each pause can last for a few seconds to a few minutes, and this occurs many times per night [1]. SAS affects 1%-6% of adults and 2% of children [7]. It affects males approximately twice as often as females [7]. Although people can be affected at any age, SAS occurs most commonly among those aged 55-60 years [7]. The prevalence of SAS is highly underestimated, as 80% of patients are undiagnosed [8]. SAS increases risks of hypertension and cardiovascular and cerebrovascular diseases. SAS also negatively affects patients’ quality of life by causing daytime sleepiness and impairing cognitive function, which can lead to accidents [8,9]. COVID-19 is a contagious coronavirus disease that was first detected in 2019, caused by SARS-CoV-2, and has led to an ongoing pandemic. As of December 23, 2020, 76 million cases and 1.7 million deaths linked to COVID-19 had been confirmed, straining health care systems throughout the world [3].

### Oxygen Saturation as a Detection and Monitoring Tool

Detecting SAS, COPD, and COVID-19 earlier in people can help reduce potential damage from these diseases, and accomplishing this requires a more thorough examination of oxygen levels. Oxygen saturation, defined as the fraction of oxygen-saturated hemoglobin relative to total blood hemoglobin, is measured either through an invasive method by sampling arterial blood to analyze the arterial oxygen saturation (SaO_2_) via a co-oximeter or with readings collected noninvasively using a pulse oximeter to measure the peripheral or pulse oxygen saturation (SpO_2_) level [10]. A pulse oximeter measures oxygen saturation in peripheral arterial blood by illuminating the skin and measuring the light absorption of oxygenated (oxyhemoglobin) and deoxygenated (reduced hemoglobin) blood using two light wavelengths: 660 nm (red) and 940 nm (infrared). The ratio of light absorbance between oxyhemoglobin and the sum of oxyhemoglobin plus deoxyhemoglobin is calculated and compared with previously calibrated direct measurements of SaO_2_ to establish an estimated measure of SpO_2_ [11,12]. Physiologically, oxygen saturation levels range from 95%-100% in a healthy person, and a decrease, also referred to as oxygen desaturation, can be suggestive of a respiratory disease [12]. Indeed, SpO_2_ levels drop to less than 90% in patients with COPD and COVID-19 as they experience extensive periods of hypoxemia and in patients with SAS when they endure repetitive oxygen desaturation events [12-14]. Although spirometry and polysomnography (PSG) are the gold standards for diagnosing COPD and SAS, respectively, these medical devices are intrusive and are not suitable for long-term monitoring and mass screening because of their high cost and lack of accessibility [15]. In the case of COVID-19, it has been observed that some patients experience oxygen levels below 90% without dyspnea, a condition termed “silent hypoxemia;” therefore, the detection of low SpO_2_ levels is even more useful for early detection and monitoring of COVID-19 [16]. Furthermore, during a pandemic that has caused a worldwide health crisis, pulse oximeters are a low-cost and easy-to-use solution to identify problems at an early stage and monitor patients at home after hospitalization [17].

### Benefits of Connected Devices

Smartwatches exhibit a high degree of satisfaction and growing popularity among the general population for health monitoring [18]. These wrist-worn devices provide a wide range of personalized health features for users, such as heart rate monitoring, sleep quality control, and oxygen saturation measurements, which can assist the prevention and long-term monitoring of diseases [19]. The Withings ScanWatch is a high-end watch with an embedded heart rate sensor that uses a reflective pulse oximeter on its caseback. The device is worn as a regular watch and is connected to a smartphone through the Withings Health Mate application. Its software, the Withings Scan Monitor, measures, transfers, records, and displays the wearer’s functional SpO_2_ level. The Withings ScanWatch is intended for intermittent measurements and can be used in hospital, sleep laboratory, long-term care, and home use environments. Therefore, the Withings ScanWatch is an alternative option to finger pulse oximeters and offers several advantages to users. It enables the user to avoid the discomfort of wearing a finger pulse oximeter (especially during sleep), and it is more firmly attached to the user’s wrist than the finger pulse oximeter, which can slip on the finger. Therefore, the Withings ScanWatch has the potential to ensure better compliance than a finger oximeter.

### Sleep Apnea

In particular, because the ScanWatch is a wrist-worn watch that can measure SpO_2_ continuously along with heart rate and, potentially, breathing rate [20,21], it could provide a less costly and time-consuming alternative to PSG. More specifically, due to the high cost of and lack of access to PSG, a subject cannot be monitored by PSG over the long term, although night-to-night variability of the apnea-hypopnea index has been found [22]. ScanWatch would enable physicians to follow patients over several weeks, at home, and in a way that does not disturb their sleep, as can occur with intrusive PSG devices. Withings ScanWatch may therefore be an alternative for screening, diagnosing, and monitoring SAS.

### Biophysics of Pulse Oximetry

**Figure 1:**
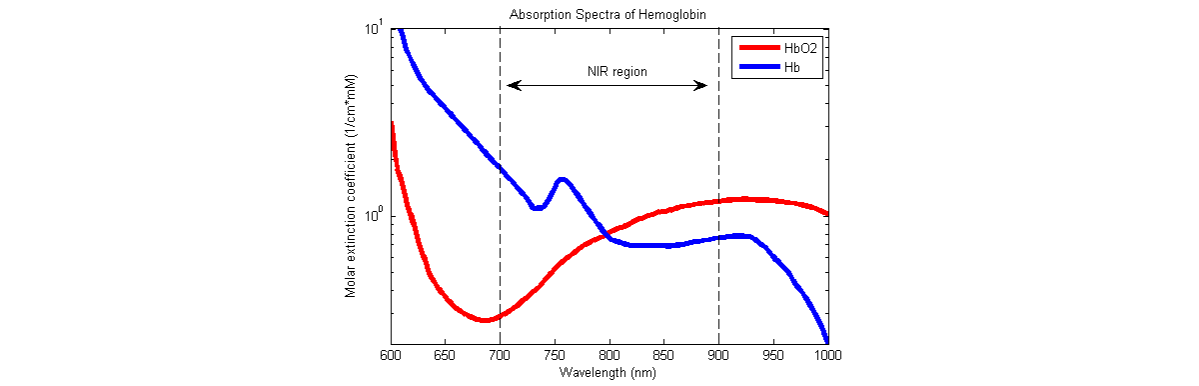
Absorption spectra of oxygenated and deoxygenated hemoglobin. Licensed from Adrian Curtin/CC BY-SA. Hb: deoxygenated hemoglobin; HbO_2_: oxygenated hemoglobin.

Pulse oximetry relies on the differential absorption by blood of red and infrared light. Figure 1 shows the absorption spectra of these two dominant forms of hemoglobin, namely oxygenated (HbO_2_) and deoxygenated (Hb).

Two wavelengths are chosen so that the ratios of the absorption rates of Hb and HbO_2_ are respectively maximal and minimal. Thus, customary choices are red light (at 660 nm) and infrared light (at 940 nm). From the intensities of the transmitted or reflected light at these two wavelengths, one can deduce the concentrations of both forms of hemoglobin, [HbO_2_] and [Hb], and the functional oxygen saturation of arterial blood (neglecting carboxyhemoglobin and methemoglobin).


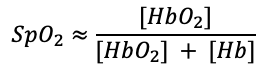


In practice, a ratio of ratios (also called modulation ratio), R, is computed as follows [23]:


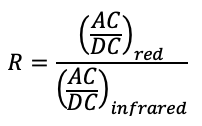


Alternating current (AC) refers to the amplitude of the pulsatile component of the PPG signal, and direct current (DC) refers to the value of its baseline. The ratio of AC/DC is commonly called the perfusion ratio.

An analytical relationship between the perfusion index R and the fraction of oxygenated hemoglobin SpO_2_ can be obtained with a model based on the Beer-Lambert law and a simplified model for the light path taken by both wavelengths. This simple model, which neglects the multiple scattering events of light in the skin, applies to some extent to pulse oximeters operating in transmission where tissues are located between the light-emitting diode (LED) and photodiode (PD). However, the model is unable to explain how reflective pulse oximeters operate, where the LED and PD are on the same side. Therefore, an experimental calibration of R to SpO_2_ during a hypoxia study is necessary.

### Reflective PPG on the Wrist and Its Challenges

Measuring SpO_2_ with a reflective sensor is more challenging on the wrist than on the fingertip. Due to the thickness of the wrist and the presence of bones, it is impossible to measure SpO_2_ in transmission. In reflectance mode, the signal-to-noise ratio (SNR) is smaller than typically found in transmission-mode finger pulse oximeters for two reasons. First, the light emitted by the LEDs penetrates the various layers of the skin, but only a small fraction will find its way back (upward) to the PD adjacent to the LED after multiple scattering events inside the skin. Second, blood perfusion is dramatically lower on the back of the wrist than on the finger. During our calibration studies, we compared blood perfusion on the wrist and on the finger using ScanWatch sensors, and we found empirically that the SNR for the measurements taken at the finger was approximately 10 times as high as the SNR for the measurements taken at the wrist.

A second challenge is that optical measurements on the wrist are more prone to artifacts. Motion artifacts are much more frequent than on the fingertip because of the presence of tendons and bones. Light-skin coupling of finger pulse oximeters is robust due to the clamp design. In the absence of a clamp, the optical sensor can lose contact with the skin more easily, both breaking the optical coupling and letting ambient light in; this tends to unpredictably modify the DC levels.

### Objectives

The main objective of this study was to clinically test and validate the accuracy of the reflective pulse oximeter function of the Withings ScanWatch to measure SpO_2_ levels during mild, moderate, and severe hypoxia compared to a co-oximeter, the ABL90 hemoximeter (Radiometer Medical ApS), in accordance with the ISO 80601-2-61:2017 standard and US Food & Drug Administration (FDA) guidance. The secondary objective was to confirm the safety of the device when used as intended.

## Methods

### Recruitment

This monocentric and prospective clinical study with a double-blind analysis was conducted on 14 healthy participants in a hypoxia laboratory at the University of California San Francisco in March 2020 at an altitude of 122 m and at a room temperature of 25 °C. Inclusion criteria were being between the ages of 18 and 50 years, having a healthy status with no evidence of any medical problems, and having both wrist circumferences between 14 and 22 cm. Current smokers, women who were pregnant, lactating, or trying to get pregnant, and participants with obesity (BMI >30 kg/m^2^) or who had an injury, deformity, or abnormality at the sensor sites and piercings that might cause air leaks during the test were excluded from the study. Participants with a known history of heart, lung, kidney, or liver disease; diabetes; clotting disorder; hemoglobinopathy or history of anemia; sensitivity to local anesthesia; or fainting or vasovagal response or any other serious systemic illness were also excluded, as well as those diagnosed with asthma, sleep apnea and Raynaud disease. Exclusion criteria also included participants with a resting heart rate of over 120 beats per minute, a systolic blood pressure over 150 mm Hg, a diastolic blood pressure over 90 mm Hg, a room air SpO_2_ under 94%, and a carboxyhemoglobin level over 3%.

### Study Design

All selected participants in the study met the inclusion criteria. Before starting the study, two Withings ScanWatch units were placed on each participant’s wrist to measure their SpO_2_, while a 22-gauge catheter was placed in their left radial artery to measure their SaO_2_. In addition, two reference finger pulse oximeters were placed on each participant to facilitate the identification of plateaus and any discrepancies between the hands. Each participant was then asked to lie in a semisupine position, to remain still, and to breathe a mixture of gas through a mouthpiece while a nose clip blocked their nose. After a 5-minute rest period, they were instructed to hyperventilate 2-3 times deeper and faster than normal during the runs to speed alveolar gas equilibration. The target SpO_2_ at each run was chosen to be evenly balanced over the 70%-100% SpO_2_ range.

**Figure 2:**
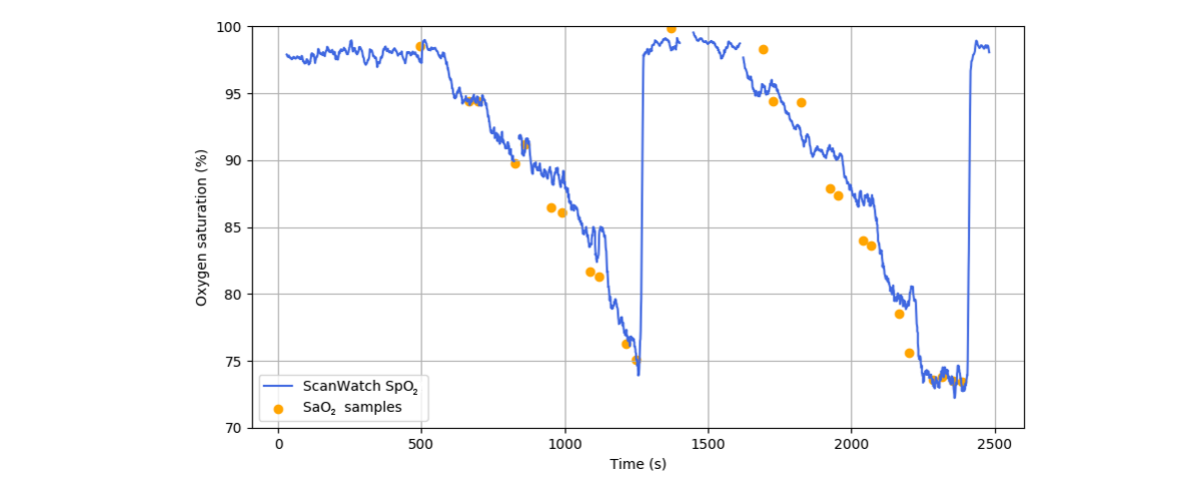
Comparison of SpO_2_ measured by the ScanWatch and hemoximeter for one subject. SpO_2_: pulse oxygen saturation as measured by pulse oximetry.

On the initial step of the run, two blood samples of 1-2 mL were first collected 30 seconds apart from the participant at the same oxygen saturation level as the room air. Then, approximately 10 seconds later, the inspired oxygen was abruptly changed to reduce oxygen saturation to the next SpO_2_ target level. On the next steps of the run, two blood samples were collected 30 seconds apart from the participant when the reference finger pulse oximeters both measured a stabilized level of oxygen saturation, with less than 1% of difference for 35 seconds. Note that the second sampling was not performed if the oxygen saturation level was destabilized between measurements. Then, approximately 10 seconds later, inspired oxygen was abruptly changed again to reach the next SpO_2_ target level. Overall, these 75-second periods of stable oxygen saturation were defined as “plateaus,” and every participant was subjected to 5 plateaus (typically 92%, 87%, 82%, 77%, and 70%) before being brought back to a high oxygen saturation level (100% O_2_) by breathing oxygen-enriched air for 2 minutes. After this run was repeated a second time, the collected blood samples (approximately 20-25 samples per participant) were immediately analyzed with the co-oximeter (ABL90 multiwavelength hemoximeter), then compared to the data recorded with the Withings ScanWatches (Figure 2).

### Registration

The study was registered at ClinicalTrials.gov (NCT04380389).

### Materials

#### General Description of the Withings ScanWatch

**Figure 3:**
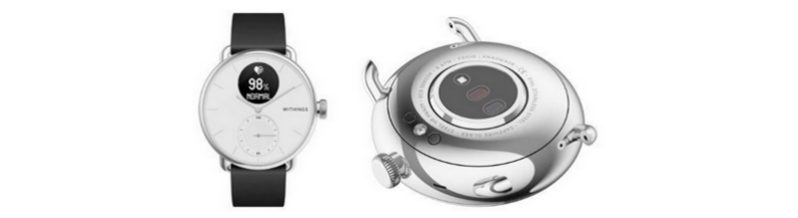
Front view (left) and back view (right) of the Withings ScanWatch.

The Withings ScanWatch is an analog battery-operated watch consisting of (1) a metal case with a connected movement, (2) three hands, with two hands indicating the time and one hand indicating a cumulated activity level, (3) an adjustable silicone band to fit any user’s wrist between 14 and 22 cm, and (4) a reflective pulse oximeter composed of three LEDs (red, infrared, and green), one broadband photodiode, and one infrared-cut photodiode (Figure 3). It includes all the necessary hardware to record and transmit SpO_2_ measurements via Bluetooth to the Health Mate application (available on Android version 6 or later and iOS version 10 or later). For the purposes of this clinical study, instead of using the original software, which displays only a single value of SpO_2_ after a 30-second measurement, we used a derived version of the Withings Scan Monitor to continuously compute, display, and record SpO_2_ levels. All the other aspects of the Withings Scan Monitor, such as its algorithm and averaging window, remained identical to its original version.

#### Design of the ScanWatch Pulse Oximeter Sensor

In a reflective design, the positions of the emitters and receptors must be carefully chosen to maximize the SNR for both wavelengths. This is achieved with optical simulations using Monte Carlo methods [24]. On average, rays detected by the photodiode follow a banana-shaped path, the depth of which depends on the wavelength [25]. On the one hand, simulations show that for a given wavelength, a higher PD-LED spacing will enable the light to pass through deeper tissues, which are more likely to be pulsatile than the superficial skin. Therefore, a longer and deeper path should increase the pulsatile modulation (or perfusion ratio) of the light signal and therefore improve the SNR.

On the other hand, increasing the LED-PD distance also comes with disadvantages. Because only a small fraction of the total light emitted by the LEDs actually reaches the photodiode, increasing this distance requires a higher light intensity, which consumes more energy. In addition, LEDs have a maximum current they can sustain without damage, so it becomes counterproductive to increase the LED-PD spacing above a certain value at which too little light would be received by the photodiode to provide a usable signal. Based on this trade-off, we found that spacings between 7 and 9 mm were optimal.

#### Algorithm

The Withings ScanWatch embeds a real-time self-contained algorithm implemented in C language designed to estimate SpO_2_. The machine learning components of the algorithm (the neural network and linear regressions) were trained using two hypoxia calibration studies, totaling 34 subjects. This algorithm comprises three parts, as described below.

First, a signal processing part estimates AC and DC for each wavelength as well as the modulation ratio. DC levels are obtained by applying a moving average filter on the raw signals, and AC levels are obtained by filtering the raw signals with a bandpass filter centered around 1 Hz and computing the moving standard deviation on the resulting signals. Thus, the perfusions (AC/DC ratios) can be computed for each wavelength, and the modulation ratio (perfusion in the red divided by the perfusion in the infrared) can be estimated. This modulation ratio is supposed to be linearly correlated with SpO_2_; however, in the case of wrist-worn oximeters, the frequent occurrence of movement and respiratory artefacts often cause that ratio to be unreliable.

To counteract the problem of the modulation ratio not being linearly correlated with SpO_2_, a second SpO_2_ estimator is used independently from the first part using a 1D convolutional neural network [26]. The network takes as an input an 8-second window over the three LED signals and outputs a direct SpO_2_ estimation. The green LED signal is provided to the network to help the network denoise the red and infrared channels. The advantage of this method over the standard one is that the advanced filter bank built inside the network can be trained to better handle movement and respiratory artefacts present on the signals, which are particularly present in reflective PPG measurements.

Finally, the modulation ratio computed in the first part and the SpO_2_ estimation calculated in the second part are merged via a linear regression to obtain a final SpO_2_ estimation.

Several indicators were used to determine whether a SpO_2_ measurement was conclusive or inconclusive. First, two algorithms use the PPG and accelerometer signals to assess if the watch is worn and if the user is still. These algorithms are heuristics that rely on simple filtering and thresholding, and they were calibrated on separate data sets acquired specifically for this purpose. The stillness of the user was derived from the absence of variation in the accelerometer signal, and the presence of a pulse on the PPG signal was the main factor to determine that the watch was worn.

In parallel, a second neural network with the same topology (8-second window over the three LED signals) detected and eliminated signals of poor quality. The calibration hypoxia studies were manually annotated to provide labels on which to train the neural network.

An SpO_2_ measurement was considered to be inconclusive if the watch was not worn, the user was moving, or the measurement was classified as being of poor signal quality.

### Statistical Analysis

A statistical analysis of the collected data was performed with the software Python 3.6.9 on a frozen database. A separate analysis was conducted for each wrist. The bias (mean error) and root mean square error (RMSE) between the SpO_2_ and SaO_2_ values were calculated for each range of SpO_2_ values (SpO_2_≤80%, 80%<SpO_2_≤90%, and 90%<SpO_2_) and for the whole range of 70%-100%. We used the Pearson correlation coefficient to determine the strength of the association between the SpO_2_ values collected from the Withings ScanWatch and the ABL90 hemoximeter. We used Bland-Altman plots to measure the agreement between the Withings ScanWatch and the ABL-90 hemoximeter.

Because a time offset exists between the Withings ScanWatch and the blood sample measured by the co-oximeter, due in part to physiological considerations (eg, distance between the arms, the wrists, and the depth of the arteries) and in part to the delay inherent to the Withings ScanWatch algorithm, we applied a plateau-matching algorithm in accordance with the recommendations of the ISO 80601-2-61:2017 standard before comparing the readings. We used a cross-correlation method to determine the delay between the measurements collected by the devices on each patient’s wrists and the blood samples. Table 1 provides an overview of the mean, median, and standard deviation values of the time offsets applied on the Withings ScanWatch measurements for plateau matching.

**Table 1:** Time offsets applied on the Withings ScanWatch for plateau matching. A negative offset indicates that Withings ScanWatch lags behind the co-oximeter.

Offset	Mean (s)	Median (s)	SD (s)
Right hand	–4.2	–4.5	9.5
Left hand	–5.2	–6.5	10.9

## Results

### User Statistics

The 14 participants in our study included 8 men and 6 women aged 23-39 years with various skin tones: fair, medium, and dark skin (Table 2). Their body mass index and blood pressure values before and after the study are also reported in this table.

**Table 2:** Demographic data of the study participants.

Subject	Age (years)	Gender	Skin pigmentation	BMI (kg/m^2^)	BP1^a^ (mm Hg)	BP2^b^ (mm Hg)
1	25	Male	Dark	22.2	91/73	111/72
2	26	Male	Medium	22.0	119/63	112/67
3	23	Female	Dark	20.8	125/63	112/67
4	23	Female	Medium	23.0	112/57	-
5	26	Female	Light	21.7	123/61	126/74
6	26	Male	Medium	28.1	113/67	120/65
7	25	Male	Medium	22.7	117/57	121/73
8	39	Male	Light	28.1	106/71	112/81
9	28	Female	Light	22.7	107/63	101/67
10	26	Male	Medium	22.4	108/64	110/55
11	28	Female	Medium	25.4	127/70	111/67
12	30	Male	Medium	20.8	138/91	132/88
13	28	Male	Dark	23.5	128/70	120/68
14	26	Female	Light	25.4	109/60	107/70

^a^BP1: blood pressure before the study.

^b^BP2 : blood pressure after the study.

### Signal Quality

Of the 322 oxygen saturation measurements collected by the Withings ScanWatches placed on the participants’ right and left wrists, 275 (85.4%) and 244 (75.8%) samples, respectively, were classified as conclusive measurements and were subsequently included for further data analysis. The remaining samples, 47/322 (14.6%) and 78/322 (24.2%) taken from the participants’ right and left wrists, respectively, were classified as inconclusive measurements (poor signal quality, motion detected, or watch not worn) and were excluded.

### Oxygen Saturation

The SpO_2_ levels collected from the conclusive measurements ranged from 70% to 100% and were evenly distributed into 3 groups: SpO_2_≤80%, 80%<SpO_2_≤90%, and 90%<SpO_2_ (Table 3). In the SpO_2_≤80%, 80%<SpO_2_≤90%, and 90%<SpO_2_ groups, we found biases of 0.75%, 2.02%, and 0.14% and RMSEs of 3.29%, 3.24%, and 2.41%, respectively, when the Withings ScanWatch was placed on the participants’ right wrists, and biases of 2.25%, 2.41%, and 0.31% and RMSEs of 3.74%, 3.21%, and 1.90%, respectively, when the Withings ScanWatch was placed on the participants’ left wrists. Overall, we found RMSEs of 3.00% and 2.97% and a bias of 0.98% (95% CI 0.65-1.32) and 1.56% (95% CI 1.24-1.87) from the participants’ right and left wrists, respectively (Table 4).

**Figure 4:**
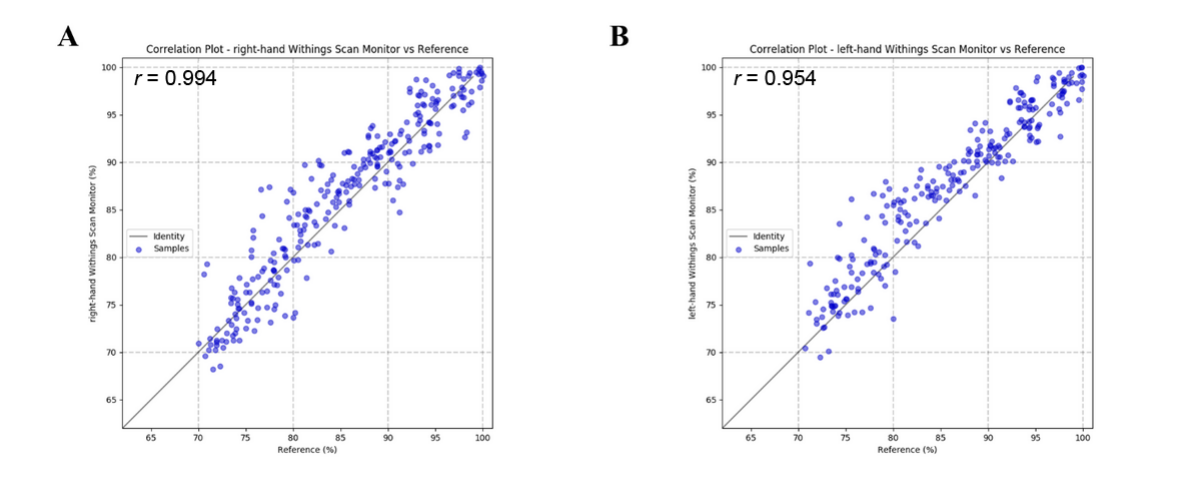
Correlation plots for the Withings ScanWatches versus the ABL90 hemoximeter from the participants’ right (A) and left (B) hands.

**Figure 5:**
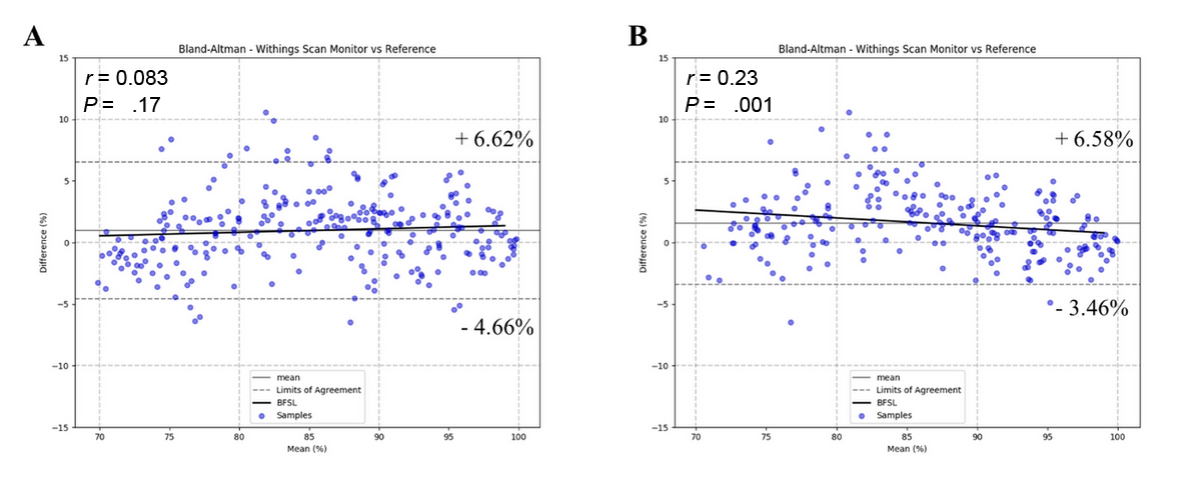
Bland-Altman plots for the Withings ScanWatches and the ABL90 hemoximeter from the participants’ right (A) and left (B) hands. BFSL: best fit straight line.

The correlation plots show a positive strong correlation between the SpO_2_ values collected from the participants’ right and left hands, with high Pearson correlation coefficients of *r*=0.944 and *r*=0.954, respectively (Figure 4). The Bland-Altman plots show a high level of agreement, with Pearson correlation coefficients of *r*=0.083 (*P*=.17) and *r*=0.23 (*P*=.001) for the devices on the right and left wrists, respectively, and 95% lower and upper limits of agreement of –4.66% to 6.62% and –3.46% to 6.58%, respectively (Figure 5).

**Table 3:** Distribution among the SpO_2_ groups of the conclusive measurements collected by the Withings ScanWatch. The corresponding distribution of SaO_2_ values given by the ABL90 hemoximeter are also reported.

Group	Values, n (%)		
	Withings ScanWatch (SpO_2_^a^)		ABL90 hemoximeter (SaO_2_^b^)
	Right wrist (n=275)	Left wrist (n=244)	Blood samples (n=322)
SpO_2_≤80%	87 (31.6)	72 (29.5)	103 (32.0)
80%<SpO_2_≤90%	95 (34.5)	78 (32.0)	109 (33.9)
90%<SpO_2_	93 (33.8)	94 (38.5)	110 (34.2)

^a^SpO_2_: pulse oxygen saturation as measured by pulse oximetry.

^b^SaO_2_: arterial oxygen saturation.

**Table 4:** Bias and RMSE found from the Withings ScanWatches placed on the participants’ right and left wrists.

Group	Values (%)			
	Right wrist		Left wrist	
	Bias	RMSE^a^	Bias	RMSE
SpO_2_≤80%	0.75	3.29	2.25	3.75
80%<SpO_2_≤90%	2.02	3.24	2.41	3.21
90%<SpO_2_	0.14	2.41	0.31	1.90
Total	0.98	3.00	1.56	2.97

^a^RSME: root mean square error.

### Safety

No undesirable effects or adverse events were reported during the study.

## Discussion

### Principal Results

Since 2014, the popularity of smartwatches has grown considerably, particularly in the health care and biomedical industries [18]. With their integrated biosensors, these wrist-worn devices have the potential to provide valuable health care data to users, thus opening new opportunities for clinical applications. Indeed, instead of going to medical facilities, which is time-consuming, costly, and requires highly trained personnel, users can now monitor their physiological conditions themselves and report any abnormalities to physicians [18,19]. The Withings Scan Monitor is the software embedded in the Withings ScanWatch, a smartwatch that displays a reflective pulse oximeter function to measure, transfer, record, and display SpO_2_ levels. Here, we tested this functionality according to the ISO 80601-2-61:2017 standard and FDA guidelines to validate its accuracy in measuring SpO_2_ levels at a clinical-grade level [27,28]. To this end, we compared the performance of the Withings ScanWatch with that of the ABL90 hemoximeter, a co-oximeter routinely used in the medical field for measuring SaO_2_ levels in blood samples, to measure the SpO_2_ levels of 14 participants who underwent plateaus of oxygen desaturation in the laboratory.

In this study, we observed an inequality in the distribution of the 325 measurements collected, with a higher rate of inconclusive measurements taken from the participants’ left wrists (78/322, 24.2%) than from their right wrists (47/322, 14.6%). This imbalance was mainly caused by unwanted movements when taking blood from the participants’ left arms. We also observed that the interactions between the catheter and the device on the left wrist interfered with the collection of the conclusive measurements when SpO_2_ levels were below 90% (Table 3). Indeed, the distribution of the samples was slightly unbalanced, with 38% of measurements collected in the 90%<SpO_2_ group. However, this difference was not significant. Thus, the ratio between the measurements distributed among the three SpO_2_ groups is acceptable. Overall, we obtained sufficient conclusive measurements to meet one of the requirements set by the ISO 80601-2-61:2017 standard and FDA guidelines, which requires at least 200 paired samples from 10 participants to validate the reflective pulse oximeter function of a device.

Next, we examined the strength of the association and the agreement between the conclusive measurements taken from the reflective pulse oximeter function of the Withings ScanWatch and the ABL90 hemoximeter. We found high Pearson correlation coefficients of *r*=0.944 and *r*=0.954 on the correlation plots and *r*=0.083 (*P*=.17) and *r*=0.23 (*P*=.001) on the Bland-Altman plots, as well as RMSEs of 2.97% and 3.00% on the participants’ right and left hands, respectively (Figure 4 and Figure 5). Taken together, these results indicate a strong association and a high level of agreement between the measurements collected from the devices. Therefore, we have demonstrated that the reflective pulse oximeter function of the Withings ScanWatch is adequate to measure the SpO_2_ levels at different stages of hypoxia.

### Detection of Sleep Apneas

**Figure 6:**
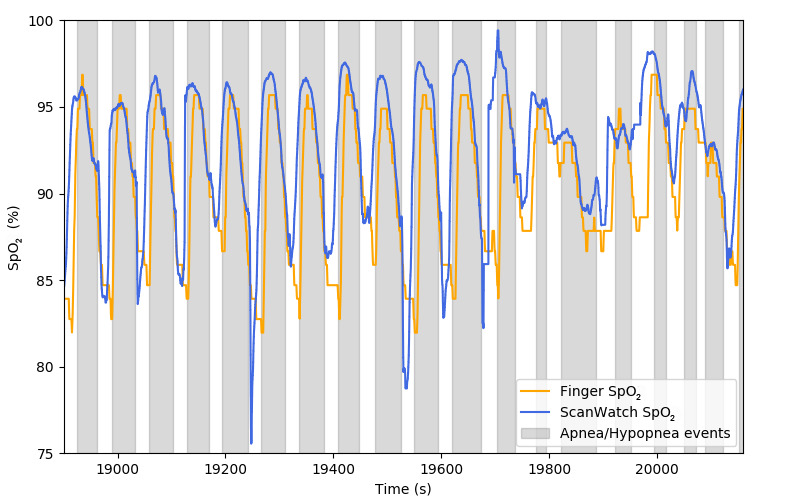
SpO_2_ measured by ScanWatch and a finger pulse oximeter during apnea/hypopnea events.

In addition to the accuracy of 3% found in the hypoxia study, the ScanWatch SpO_2_ algorithm possesses adequate resolution and dynamics to identify apnea and hypopnea events when worn during sleep (Figure 6). Indeed, when used in a continuous monitoring mode, the ScanWatch can detect short SpO_2_ variations that occur in apneic patients, paving the way for automatic sleep apnea detection for patients at home without using intrusive and costly polysomnography setups.

### Limitations

The clinical study was conducted in a controlled environment with a well-established protocol and methodology to collect SpO_2_ measurements during stable plateaus of SpO_2_ in healthy participants aged 23-39 years. Its design may limit the generalizability of the results to real-world situations. Indeed, the ability of the Withings ScanWatch reflective pulse oximeter to dynamically monitor the evolution of SpO_2_ in a subject is unknown in the real world because participants were exposed to stable plateaus of SpO_2_ between 70% and 100% in this study. Finally, given the risks induced by hypoxia on older subjects or patients with respiratory conditions, we were unable to test the ability of the Withings ScanWatch to measure SpO_2_ in these populations. In future work, the accuracy of the Withings ScanWatch reflective pulse oximeter should therefore be tested in real-life conditions (including in the home, at a hospital ward, and during rehabilitation), on a specific population such as patients with COPD or obstructive sleep apnea, or to diagnose and monitor patients with respiratory diseases.

### Conclusions

FDA guidance and the ISO 80601-2-61:2017 standard require RMSEs below 3.5% and 4% for reflectance pulse oximeter approval, respectively [27,28]. These criteria were recently fulfilled by a wrist-sensor pulse oximeter, the Oxitone 1000, in a study in which its precision and accuracy were tested and an RMSE of 3% was reported [29]. Here, out of the conclusive measurements collected from the 14 participants, we have shown that the Withings ScanWatch exhibited acceptable RMSE levels for SpO_2_ that were below the thresholds defined by these authorities. According to our data, we have thereby demonstrated that the Withings ScanWatch fulfills the requirements set by both the FDA guidelines and the ISO 80601-2-61 standard. The reflective pulse oximeter function of the Withings ScanWatch is thus validated and is accurate in measuring SpO_2_ levels at a clinical-grade level. No undesirable effects or adverse events were reported during the study.

## Abbreviations

AC: alternating current
COPD: chronic obstructive pulmonary disease
DC: direct current
FDA: US Food & Drug Administration
Hb: deoxygenated hemoglobin
HbO_2_: oxygenated hemoglobin
LED: light-emitting diode
PD: photodiode
PPG: photoplethysmography
PSG: polysomnography
RMSE: root mean square error
SaO_2_: arterial oxygen saturation
SAS: sleep apnea syndrome
SNR: signal-to-noise ratio
SpO_2_: pulse oxygen saturation as measured by pulse oximetry
